# A quantization algorithm of visual fatigue based on underdamped second order stochastic resonance for steady state visual evoked potentials

**DOI:** 10.3389/fnins.2023.1278652

**Published:** 2023-11-21

**Authors:** Peiyuan Tian, Guanghua Xu, Chengcheng Han, Xun Zhang, Xiaowei Zheng, Fan Wei, Sicong Zhang, Zhe Zhao

**Affiliations:** ^1^School of Mechanical Engineering, Xi’an Jiaotong University, Xi’an, China; ^2^State Key Laboratory for Manufacturing Systems Engineering, Xi’an Jiaotong University, Xi’an, China; ^3^The First Affiliated Hospital of Xi’an Jiaotong University, Xi’an, China; ^4^School of Microelectronics, Xi’an Jiaotong University, Xi’an, China

**Keywords:** SSVEP, visual fatigue, quantification algorithm, underdamped second-order stochastic resonance, fixed step-energy parameter optimization algorithm

## Abstract

**Introduction:**

In recent years, more and more attention has been paid to the visual fatigue caused by steady state visual evoked potential (SSVEP) paradigm. It is well known that the large-scale application of brain-computer interface is closely related to SSVEP, and the fatigue caused by SSVEP paradigm leads to the reduction of application effect. At present, the mainstream method of objectively quantifying visual fatigue in SSVEP paradigm is based on traditional canonical correlation analysis (CCA).

**Methods:**

In this paper, we propose a new SSVEP paradigm visual fatigue quantification algorithm based on underdamped second-order stochastic resonance (USSR) to accurately quantify visual fatigue caused by SSVEP paradigm in different working modes using single-channel electroencephalogram (EEG) signals. This scheme uses the fixed-step energy parameter optimization algorithm we designed, combined with the USSR model, to significantly improve the signal-to-noise ratio of the processed signal at the target characteristic frequency. We not only compared the new algorithm with CCA, but also with the traditional subjective quantitative visual fatigue gold standard Likert fatigue scale.

**Results:**

There was no significant difference (*p* = 0.090) between the quantitative value of paradigm fatigue obtained by the single channel SSVEP processed by the new algorithm and the gold standard of subjective fatigue quantification, while there was a significant difference (*p* < 0.001^***^) between the quantitative value of paradigm fatigue obtained by the traditional multi-channel CCA algorithm and the gold standard of subjective fatigue quantification.

**Discussion:**

The conclusion shows that the quantization value obtained by the new algorithm can better match the subjective gold standard score, which also shows that the new algorithm is more reliable, which reflects the superiority of the new algorithm.

## 1 Introduction

Brain-computer interface has now become a hot direction. One of the main directions of brain-computer interface is to allow patients to communicate with the outside world through paradigm stimulation, using only the head, such as brain-computer spelling (Han et al., 2018), brain-controlled wheelchair (Diez et al., 2013) applications, etc. The paradigm used in these applications is the SSVEP paradigm, but in actual use, the SSVEP paradigm will cause obvious visual fatigue, which will reduce the information transfer rate, thereby reducing the use effect of the application, and even cause serious consequences such as reading errors (Cao et al., 2014; Zhao et al., 2021; Zhu et al., 2021; Zhang et al., 2022). At the same time, epidemiological studies have shown that as many as 90% of digital display users have varying degrees of visual fatigue (Coles-Brennan et al., 2019). Therefore, fatigue detection based on SSVEP paradigm is particularly important. Traditional visual fatigue detection includes subjective fatigue scale detection (Guo et al., 2021), subjective and objective [eye movement (Xie et al., 2021) and EEG (Lee et al., 2021)] combined visual fatigue detection, etc. In recent years, as visual fatigue is closely related to brain state, relevant studies have also pointed out that measuring brain state can also evaluate visual fatigue to a certain extent (Li et al., 2017, 2023; Li W.-K. et al., 2022). Here, nonlinear algorithms are widely used (Li R. et al., 2022), which also provides ideas for the algorithm in this paper.

Many previous visual fatigue assessment methods are based on subjective fatigue scales. In many cases, the scores of these subjective scales are regarded as the ‘gold standard’ (Heuer et al., 1989; Hayes et al., 2007). From an objective point of view, the existing fatigue quantitative methods, such as critical flicker frequency (Benedetto et al., 2013; Liu et al., 2021), EEG (Lee et al., 2021), and eye movement (Feng and Chen, 2021; Sugimoto et al., 2022), have a certain effect. Among them, CCA is often used to qualitatively or quantitatively analyze the fatigue of EEG signals. CCA is a multivariate statistical analysis method, which uses the correlation between variable pairs to reflect the overall correlation between the two groups of indicators. In order to grasp the correlation between the two sets of indicators as a whole, two linear projection vectors (a linear combination of each indicator in the two groups) were extracted from the two sets of indicators, and the correlation between the two vectors was used to reflect the total correlation between the two sets of indicators. At present, the commonly used indicators for quantitative analysis of visual fatigue in EEG are the amplitude of CCA, the signal-to-noise ratio of CCA, and the energy band of CCA (Xie et al., 2016; Zheng et al., 2020). Not only for the SSVEP paradigm, in the objective detection of various types of visual fatigue, researchers use the test data to perform some simple and objective quantitative analysis of visual fatigue. However, due to the lack of recognized objective gold standards and related evaluation indicators in academia, these quantitative analyzes lack comparability, and it is difficult for researchers to compare the advantages and disadvantages of these quantitative methods (Bier et al., 2020; Hamedani et al., 2020; Hu and Lodewijks, 2020; Zheng et al., 2020; Ali et al., 2021; Graves et al., 2022). It is found that the existing objective quantification of visual fatigue is often very small and cannot match the subjective scale score better. Meanwhile, the existing CCA algorithm is aimed at multi-channel EEG data, but now the industry urgently needs fatigue assessment for single-channel EEG, such as fatigue driving test which is mainly because single-channel detection is low in cost, easy to operate, and easy to popularize (Wang Y. Q. et al., 2022). However, single-channel data are often not obvious enough (Ogino et al., 2019) and need to be strengthened. Therefore, the algorithm in this paper has two purposes. One is to process only the single-channel SSVEP signal from the perspective of cost saving, the other is to improve the signal-to-noise ratio of the processed signal from the perspective of improving energy utilization, so as to improve the accuracy of objective quantification of SSVEP paradigm fatigue. It not only makes the degree of objective quantification more consistent with the subjective scale score, but also makes the objective quantitative results more convincing, and better reflects the degree of visual fatigue of the subjects from an objective point of view, which is helpful to formulate the ‘gold standard’ of objective visual fatigue. In summary, this paper proposes a new evaluation model for detecting and quantitatively analyzing single-channel EEG signals based on underdamped second-order stochastic resonance noise enhancement for visual fatigue caused by SSVEP paradigm.

The nonlinear system comprises second-order stochastic differential equations is called the underdamped second-order stochastic resonance model. When the damping is small, the output signal has a large random fluctuation. At this time, the random interference of noise plays a leading role, and the burr of the signal is large. With the increase of damping, the fluctuation component in the output signal is gradually squeezed, and the response of the system is enhanced. However, exorbitant damping will make the system output state incapable to keep up with the response speed of the input signal during the transfer process. At the same time, the amplitude of the noise and the driving signal is also greatly filtered out, resulting in distortion of the output signal. Therefore, for different input signals, there will be an optimal damping coefficient, which makes the underdamped second-order stochastic resonance system have the best filtering effect (Yao et al., 2019). The EEG signal of the light scintillation paradigm will respond at the characteristic frequency and multiple harmonic frequencies (Liu et al., 2022). However, if researchers want to evaluate visual fatigue, multiple responses will interfere with the analysis results. Therefore, in order to improve recognition accuracy, it is necessary to study a feature frequency extraction technique that uses noise energy to highlight useful information. Underdamped second-order stochastic resonance uses noise energy to enhance weak signals and suppress noise without damaging useful signals. Its output frequency response is amount to a set of nonlinear band-pass filters, which is suitable for extracting SSVEP.

Next, the second part of the article details the visual fatigue quantitative assessment algorithm we use. The third part introduces the source of the analog model and the real model and uses the model to verify and compare the algorithm. The fourth part discusses the advantages and limitations of several algorithms and possible future improvements. The fifth part summarizes the performance of USSR.

## 2 Materials and methods

### 2.1 Quantization algorithm

#### 2.1.1 Fixed step size energy visual fatigue quantification algorithm based on underdamped second-order stochastic resonance

The brain produces fatigue after a period of activity, which is reflected in the decrease of EEG response amplitude induced by SSVEP paradigm (Zheng et al., 2020). Therefore, objective quantification of visual fatigue can start from this aspect. The traditional CCA can quantify visual fatigue to a certain extent, but due to noise interference caused by power frequency artifacts, electrode drift, etc., the energy available for visual fatigue assessment is weak and the signal-to-noise ratio is low. The new fatigue algorithm proposed in this paper is based on stochastic resonance. Stochastic resonance enhances weak signals and suppresses noise through noise resonance, improves signal-to-noise ratio, makes the signal more effective, and is more conducive to objectively quantifying visual fatigue.

The innovative precise quantization algorithm of visual fatigue in this paper is an evaluation model for single-channel EEG signals designed by combining the fixed step-energy parameter optimization algorithm with the underdamped second-order stochastic resonance model.

The differential equations corresponding to the underdamped second-order stochastic resonance model are as follows:


(1) $$\begin{array}{l} \frac{d^{2}x}{dt^{2}}=-\frac{dU\left(x\right)}{dx}-\beta \frac{dx}{dt}+s\left(t\right)+\varepsilon \left(t\right)=\mathrm{ax}-bx^{3}\\ \;-\beta \frac{dx}{dt}+s\left(t\right)+\varepsilon \left(t\right)\; \end{array}$$

In the formula: x denotes the signal to be processed; a>0,b>0 are system parameters; 0<β<1 indicates the damping factor; $s\left(t\right)=\mathrm{Acos}\left(2\pi ft+\varphi \right)$ is the input periodic excitation signal; $\varepsilon \left(t\right)$ represents Gaussian white noise. To bring the expression $s\left(t\right)$ into the above expression is:


(2) $$\frac{d^{2}x}{dt^{2}}=\mathrm{ax}-bx^{3}-\beta \frac{dx}{dt}+\mathrm{Acos}\left(2\pi ft+\varphi \right)+\varepsilon \left(t\right)$$

In the model designed in this paper, $a=1,b=1,A=1,f=0.05\mathrm{Hz},\varphi =0,\varepsilon \left(t\right)$ is white noise with a noise intensity of 2, and β is the damping factor. The step size parameter h is set to determine the compressed sampling frequency according to the sampling frequency and a certain compression ratio before adding the data to the stochastic resonance model. This compressed sampling frequency is the reciprocal of the step size parameter h. Although step size parameter h is not in Formula (2), for the underdamped second-order stochastic resonance, the equivalent center frequency and bandwidth of the output frequency response are also approximately positively correlated with the step size h of the numerical calculation. The step size of the numerical calculation determines the passband range of the stochastic resonance model. Therefore, when using stochastic resonance to extract the response amplitude of SSVEP at the characteristic frequency, it is necessary to combine the frequency range of the analysis signal to select the best step size parameter h to achieve better stochastic resonance effect. In this algorithm, according to experience and the definition of compression sampling frequency and damping factor, the step size parameter h range is set to 1/35–1/3, and the damping factor β range is set to 0.05–0.85.

The quantitative model of underdamped second-order stochastic resonance will produce different resonance results due to different step parameters and damping coefficients. These results are the data after enhancing the wanted signal and weakening the noise. In these data, it will be screened again, and the signal that is most accurate for quantifying visual fatigue is selected from all the signals that meet the resonance optimization results. Finally, following the analysis of paradigm visual fatigue by CCA algorithm (Zheng et al., 2020), the amplitude of the signal processed by this algorithm at the characteristic frequency is defined as the paradigm visual fatigue quantitative value of the subject in this specific state.

#### 2.1.2 Flow chart of the innovative algorithm

**Figure 1:**
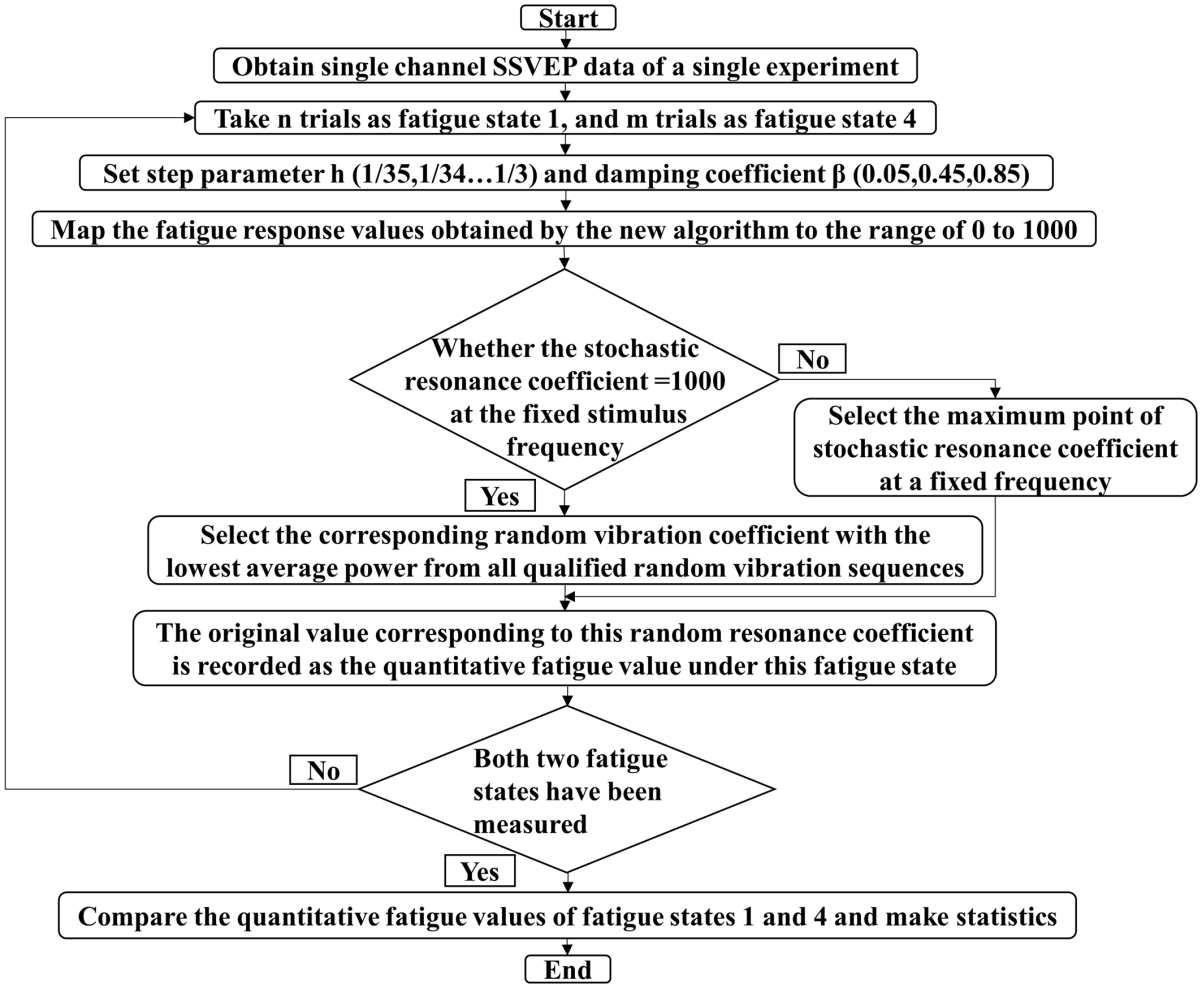
Flow chart of the innovative algorithm.

As shown in Figure 1 is the flow chart of the entire visual fatigue accurate quantification algorithm, explained as follows: At the beginning of the process, the precise quantization algorithm first pre-processes the obtained single-channel EEG signals and groups them according to the fatigue level. Then, according to the step size parameters (1/35,1/34,...,1/3) and damping coefficient (0.05,0.45,0.85) within a certain range set in advance, the obtained signals are processed, and the obtained 99 sets of stochastic resonance results are screened. Firstly, since the stochastic resonance response amplitude is mapped in the range of 0 to 1,000, it is judged whether there is a group with the largest amplitude (=1,000) at the characteristic frequency. If several groups meet the conditions, the group with the smallest average power in these groups is further screened. This group of results is the required group and goes back to the previous step. If all groups do not meet the condition that the amplitude at the characteristic frequency is the largest (=1,000), the group with the relative maximum value of the stochastic resonance coefficient at the characteristic frequency of these groups is selected as the required group. At this time, the only required group is obtained, and the stochastic resonance coefficient at the characteristic frequency of the group is recorded as the visual fatigue quantification value of the subject in a fatigue state defined by this algorithm, and the process ends.

#### 2.1.3 Quantitative algorithm of visual fatigue based on canonical correlation analysis parameters

CCA is different from the stochastic resonance processing algorithm only for single-channel EEG signals. It is a general algorithm for the multi-channel measurement of EEG indicators. CCA uses the correlation between each channel data to analyze the overall data and then judges the characteristics of EEG signals in this state.

#### 2.1.4 Statistical method

Statistical analyzes were carried out using SPSS 22.0 (IBM, Armonk, United States). The Pearson correlation test with a significance of *p* < 0.05 was employed to evaluate the Pearson correlation and significance of different indicators. Kolmogorov–Smirnov test was used to test whether the model obeys the normal distribution. Levene test with a significance of *p* < 0.05 was employed to evaluate the Homogeneity of variances of the relative indices. Kruskal-Wallis H-test with a significance of *p* < 0.05 was employed to test the statistics. Pairwise comparison with a significance of *p* < 0.05 was employed to compare the differences between several paired indicators. Besides that, the box diagram of the comparisons of five quantitative methods of visual fatigue in details is also analyzed and drawn by Origin 2018 (OriginLab, Northampton, United States).

## 3 Results

In this section, in order to verify whether the USSR can reduce the energy spillover at the characteristic frequency and improve the signal-to-noise ratio, the USSR is first tested on the analog signal model of sinusoidal signal superimposed with white noise, and its performance is compared with the original CCA method. Then, the USSR was applied to the real EEG recording in the SSVEP experiment to evaluate the performance of the method in detecting the degree of attention change of the participants.

### 3.1 Analysis of analog signal model

Since the existing fatigue detection algorithm for SSVEP paradigm is CCA, the difference between the proposed algorithm USSR and CCA in processing signals is compared. In order to verify, a signal superimposed by sinusoidal signal and white noise is simulated firstly, and then the results of using USSR and CCA are compared, respectively.

#### 3.1.1 Settings of analog signal model

The first step is signal generation.


(3) $$\begin{array}{l} y1=10*\sin\left(2*\Pi *f1*t\right)+5*\sin\left(2*\Pi *f2*t\right)+2.5*\sin\\ \;\left(2*\Pi *f3*t\right)+1.25*\sin\left(2*\Pi *f4*t\right)\; \end{array}$$

4 $$y2=A*\eta \left(t\right)$$

(5) y=y1+y2

In the above formulas, f1 = 7.5 Hz, f2 = 15 Hz, f3 = 22.5 Hz, f4 = 30 Hz, f1 represents the main frequency component, f2 , f3 , f4 represents the harmonic frequency component. A = 1, $\eta \left(t\right)$ is a random variable obeying Gaussian distribution with mean value of 0 and variance of 1.

#### 3.1.2 Comparison between USSR and CCA

**Figure 2:**
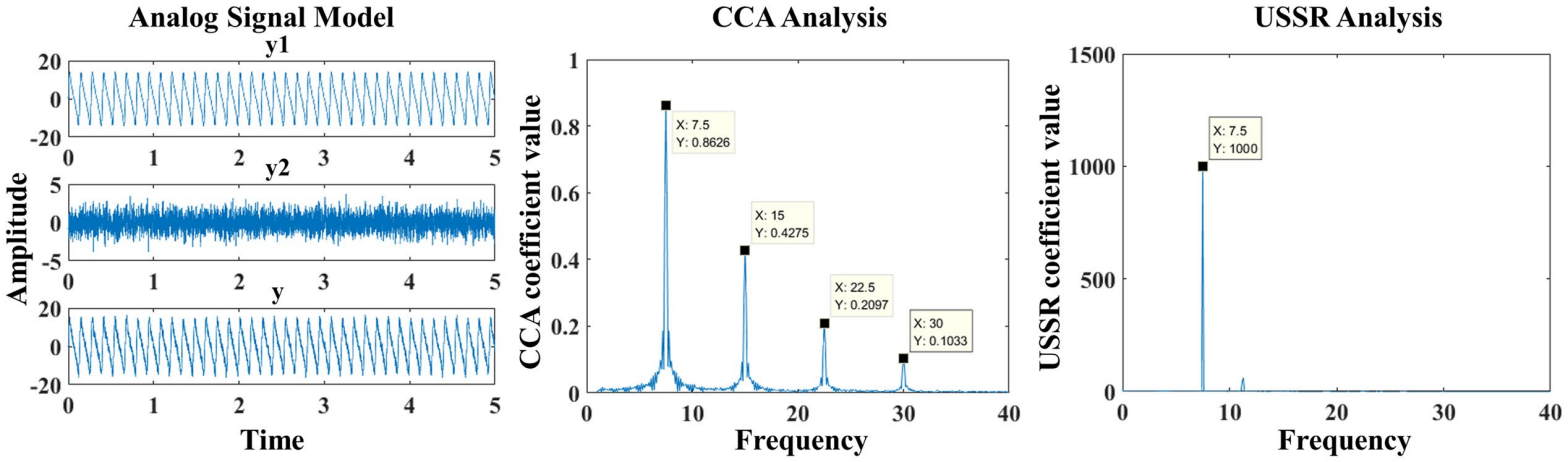
Generation of analog signals and comparison after CCA and USSR processing.

From Figure 2, it can be seen that CCA can effectively identify the four frequency components. According to previous research papers, the fatigue quantification exploration of SSVEP paradigm is based on the energy at the feature frequency of the paradigm. However, from USSR analysis, it can be seen that its energy mainly converges at the main frequency (feature frequency). In the experimental analysis only for the main frequency, USSR can effectively improve the signal-to-noise ratio, which is more conducive to the study of the changes in the attention of the subjects in the SSVEP experiment, so as to explore the changes in the visual perception of the subjects.

### 3.2 Analysis of real model

**Figure 3:**
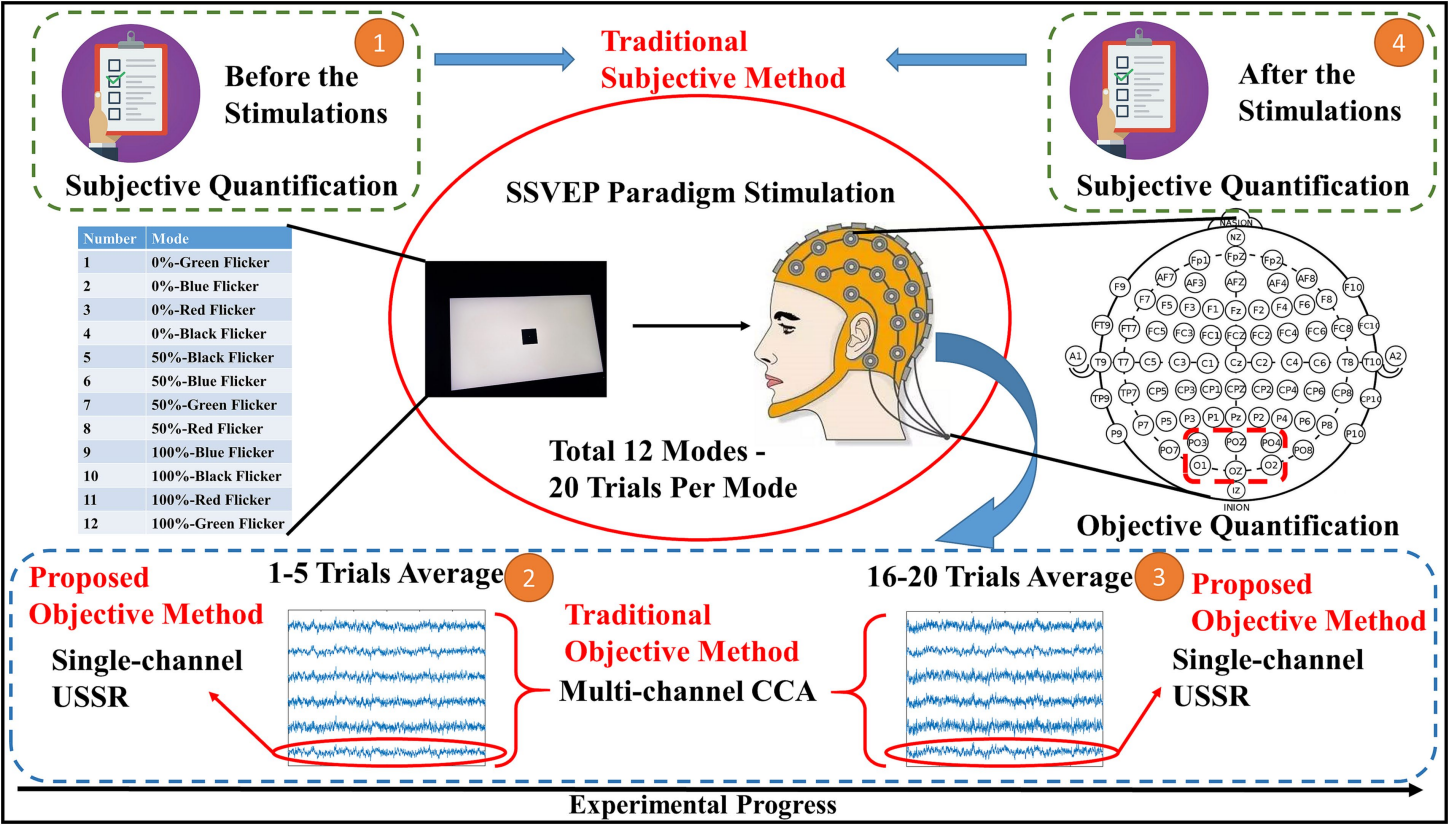
Experimental flowchart of a real model.

Figure 3 is a schematic diagram of the whole real EEG experimental model. It can be seen that the experiment consists of two parts: subjective quantification and objective quantification. According to the order, there was a subjective Likert scale measurement before the experiment, as the fatigue state value before the experiment in the subjective detection. Then the EEG experiment was started. The experimental stimulation paradigm consisted of 12 different modes of SSVEP paradigm, and there were 20 trials in each mode. Take n trials as fatigue state 1, and m trials as fatigue state 4 as Figure 1 explains. When the proposed algorithm applied to the real model, *n* represents 1 to 5 trials, and m represents 16 to 20 trials. The first five trials and the last five trials were used as the fatigue state values before and after the experiment in the objective detection. After the paradigm stimulation, a subjective scale measurement was performed again as the fatigue state value after the experiment in the subjective detection.

#### 3.2.1 Subjects of real model

All the experiment data is from the database of an existing paper (Tian et al., 2022) which we have obtained approval to use it. A total of 15 subjects were recruited, aged 26 ± 0.55 years old, without eye diseases, and all subjects had uncorrected visual acuity or corrected visual acuity above 1.0. Before the experiment, sufficient sleep and no drinking were ensured. All subjects were given informed consent according to the Helsinki Declaration and approved by the institutional review committee of Xi’an Jiaotong University.

#### 3.2.2 Experimental settings of real model

In a pure black indoor environment, the brightness percentage of the display screen was set to 0, 50, and 100%, and the color of the light flicker stimulation paradigm used to induce SSVEP was set to black, red, green, and blue. Therefore, there are total 12 different paradigm environments as shown in Table 1. The aim of setting 12 different modes is to investigate whether the quantitative indicators of visual fatigue involved in the manuscript are consistent under different modes.

**Table 1:** Twelve modes based on SSVEP paradigm.

Paradigm	Detailed settings
1	0% brightness – Green light flicker
2	0% brightness – Blue light flicker
3	0% brightness – Red light flicker
4	0% brightness – Black light flicker
5	50% brightness – Black light flicker
6	50% brightness – Blue light flicker
7	50% brightness – Green light flicker
8	50% brightness – Red light flicker
9	100% brightness – Blue light flicker
10	100% brightness – Black light flicker
11	100% brightness – Red light flicker
12	100% brightness – Green light flicker

For visual fatigue detection, according to the international 10–20 system, the electrodes were assigned to the occipital region, in PO3, PO4, POz, O1, O2, and Oz, respectively. There were a total of 12 groups of experiments. Subjects in each group were allowed to rest until fatigue was relieved. This time was controlled by themselves. To avoid other interference, the time was arranged from 8 p.m. to 10 p.m. According to the literature of others (Zheng et al., 2020), there were 20 paradigm stimuli in each group, and the paradigm flicker frequency was 7.5 Hz. Before each stimulation, the prompt was 0.5 s, the stimulation lasted for 4.0 s, and the rest was 0.5 s after the stimulation. The participants randomly selected the experimental order of the 12 groups to prevent the effect of fixed order on the detection. The sampling rate of g.tec (for EEG acquisition and processing) and Tobii Pro-fusion (for pupil information acquisition and processing) are 1,200 Hz and 120 Hz, respectively. Before the algorithm analyzes the data, an online band-pass filter with a bandwidth of 2–100 Hz and an offline notch filter with a bandwidth of 48–52 Hz are used to eliminate artifacts and power line interference, respectively.

#### 3.2.3 Comparison between USSR and CCA

**Figure 4:**
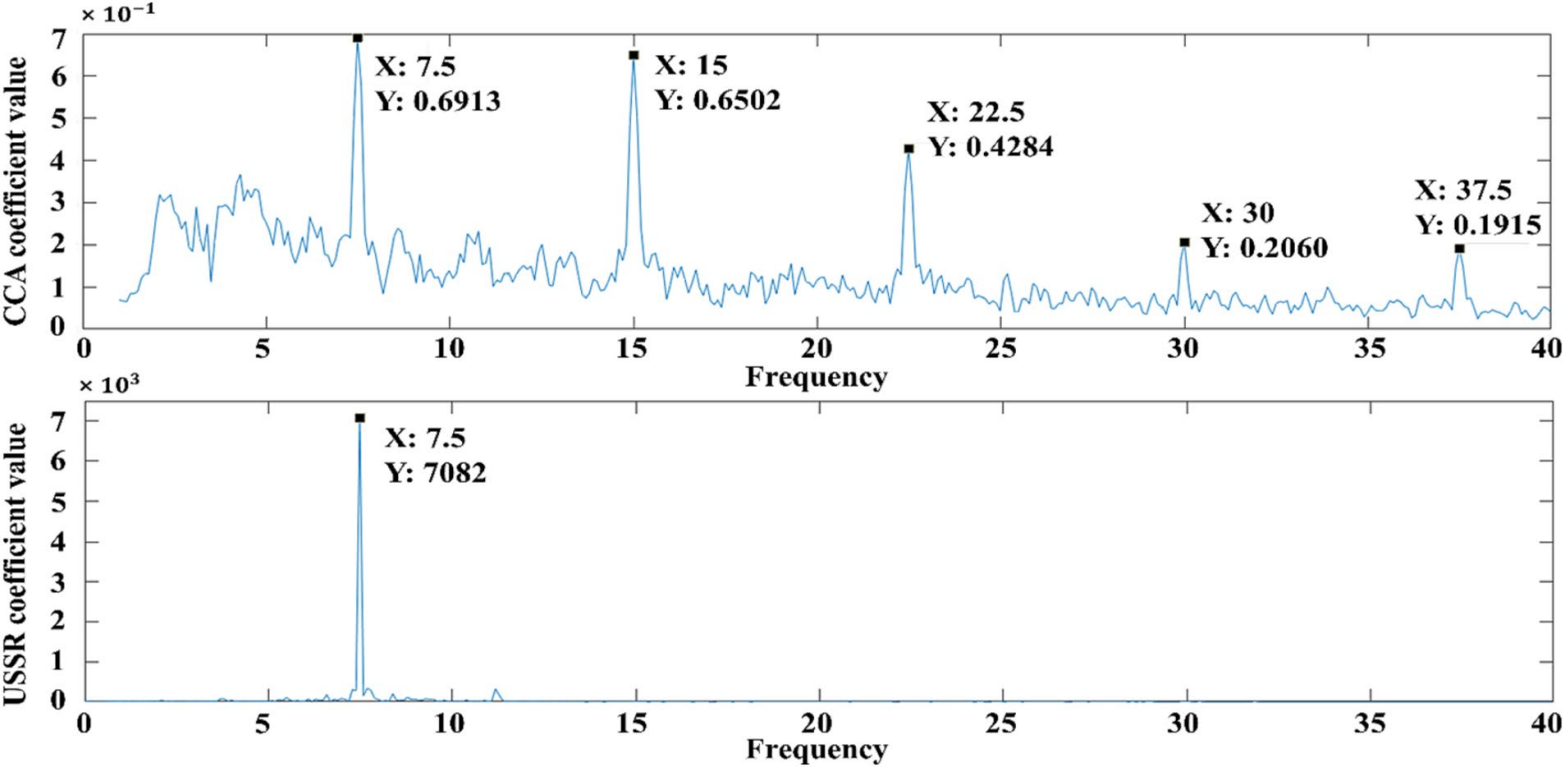
Difference between USSR and CCA in visual fatigue quantification based on EEG comparison.

Firstly, the difference between the USSR algorithm proposed in this paper and the traditional CCA algorithm in the objective quantification of visual fatigue EEG is compared. The data source of CCA and USSR are the six-channel EEG data and the single-channel (Oz) EEG data of the second experiment of subject 9, respectively. The traditional CCA is a multi-channel algorithm, and the algorithm in this paper is a single-channel algorithm, because from the perspective of this paper, the traditional multi-channel algorithm is to improve the signal-to-noise ratio. The new algorithm in this paper can not only achieve this purpose from a single channel, but also take into account the advantages of easy arrangement and low cost of a single channel. The six channels arranged in the experiment can be used alone. In this model, we selected a representative channel Oz for analysis. As can be seen from Figure 4, since the paradigm used is an internationally accepted light scintillation paradigm, it is convenient for other researchers to reproduce, but the light scintillation paradigm will induce frequency doubling characteristics other than the characteristic frequency. Using CCA analysis, it can be seen that in addition to the response at the characteristic frequency (7.5 Hz), there are strong responses at the second harmonic (15 Hz), the third harmonic (22.5 Hz), the fourth harmonic (30 Hz), and the fifth harmonic (37.5 Hz). The USSR analysis shows that there is only a strong response at the characteristic frequency (7.5 Hz).

In the objective quantification of visual fatigue, the response value at the characteristic frequency is used as the quantitative value of visual fatigue (Zheng et al., 2020). Due to the instability of the multi-frequency response, the response at the multi-frequency will cause energy overflow and waste in the CCA analysis, which will also affect the accuracy of visual fatigue quantification to a certain extent. Therefore, it is urgent to stabilize the EEG response at the characteristic frequency and minimize the energy overflow. The USSR algorithm proposed in this paper satisfies this requirement well. Using the response value at the characteristic frequency obtained by USSR analysis as the visual fatigue quantization value can greatly ensure the quantization accuracy and improve the objectivity and accuracy of visual fatigue quantization based on EEG.

**Figure 5:**
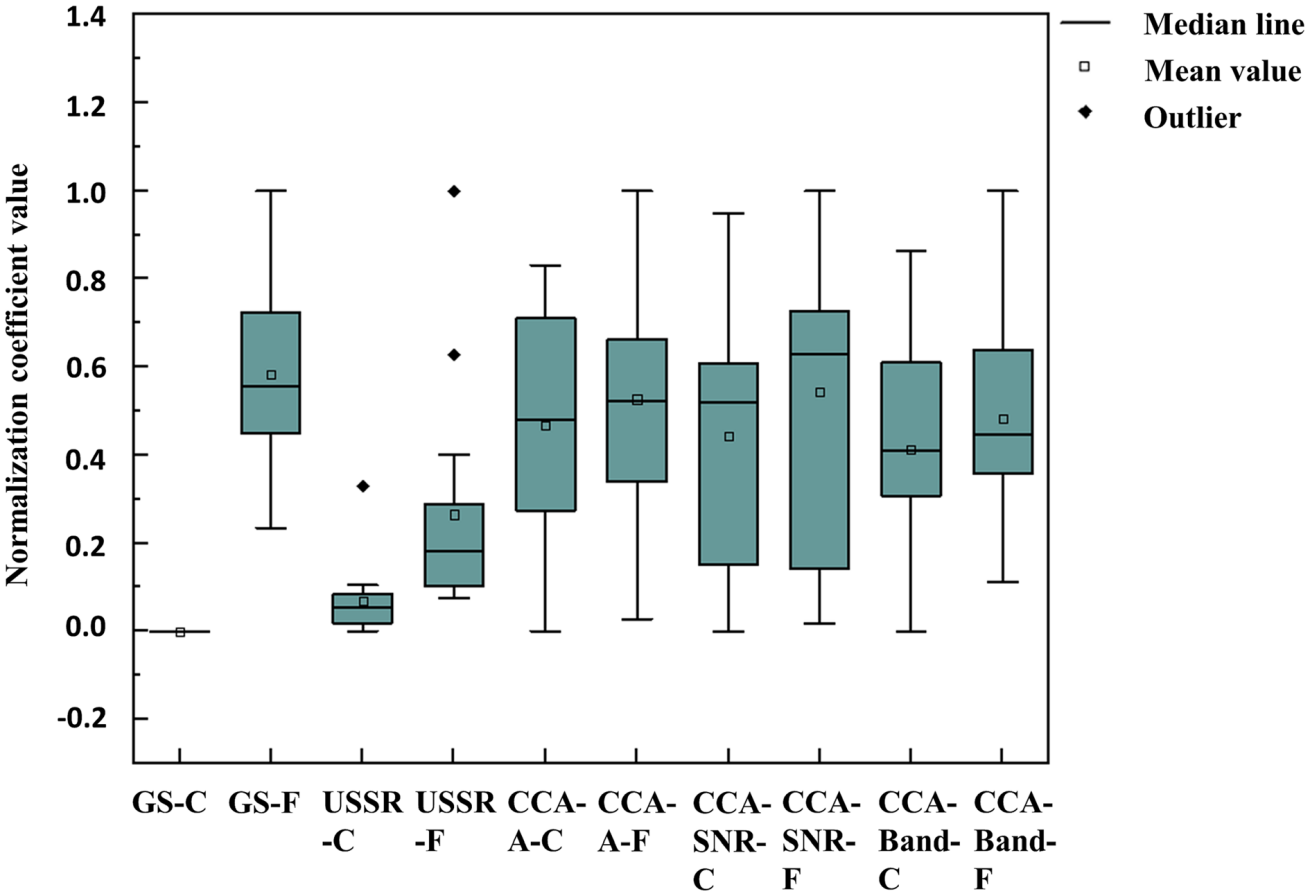
The average performance of 15 subjects in 5 visual fatigue quantification methods under 12 different paradigm environments. -C means consciousness state, -F means fatigue state.

**Figure 6:**
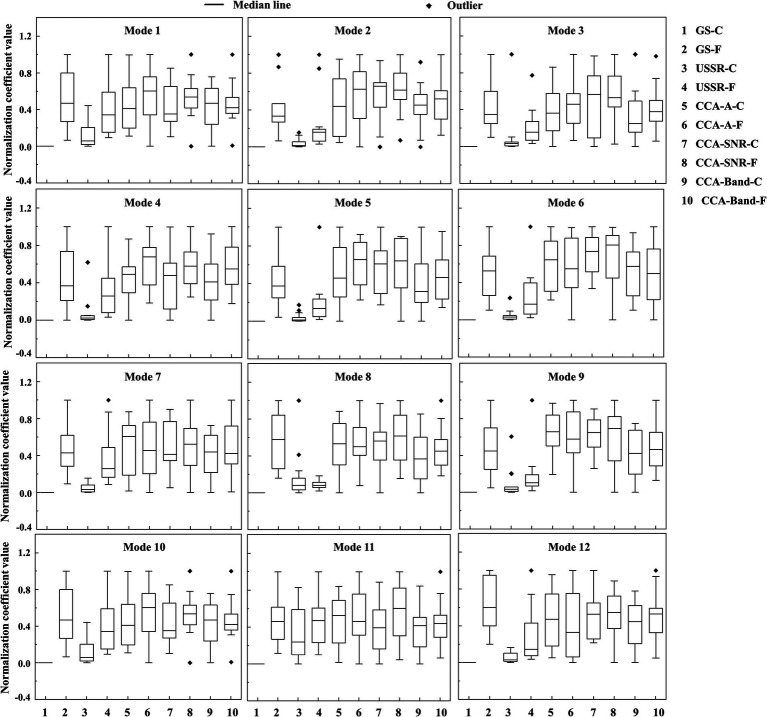
Comparison of five quantitative methods of visual fatigue in details. -C means consciousness state, -F means fatigue state.

Figures 5, 6 are the comparison box diagram of five quantitative visual fatigue methods, of which golden standard (GS) is a subjective method and the other four are objective methods. The GS here refers to the gold standard, that is, the most subjective scoring method used by the academic community to evaluate visual fatigue. GS is evaluated by the Likert scale. We score the subjects before and after each group of experiments. The pre-experiment scoring is to confirm that the subjects have rested well so that the next group of experiments can be carried out. If any of the scores before the experiment is not 1 (the easiest), we will let the subjects continue to rest their eyes until all items are scored 1, which also explains from the side why the GS-C in Figures 5, 6 are the same and the mean value is normalized to 0. The post-test scoring is to obtain the corresponding score of their visual fatigue degree from a subjective point of view, which is also one of the most mainstream methods in the academic community. Therefore, this paper takes the subjectively obtained visual fatigue assessment score as the gold standard for subsequent comparison and analysis. The Likert scale used in this experiment has been placed in the Supplementary materials.

After subtracting and dimensionless the quantization values of all algorithms in the consciousness states (-C) and fatigue states (-F), we obtained the following data results, which can also be seen from Figure 5 that among the four objective quantitative methods, only the quantitative method based on USSR (0.49 ± 0.25) is the closest to the GS (1.59 ± 0.62), and it is also superior to the other three CCA-based quantitative methods [CCA-A (0.03 ± 0.10), CCA-SNR (−0.01 ± 0.24), CCA-Band (0.08 ± 0.11)].

#### 3.2.4 Statistical test of five algorithms

The Pearson correlation test is carried out on the normalized fatigue difference obtained by the five algorithms.

It can be seen from Table 2 that the significance of the algorithm proposed in this paper (USSR) and the GS is less than 0.01, indicating that there is a linear relationship. Then the correlation coefficient is 0.724, which is between 0.5 and 0.8, showing that there is a medium correlation between USSR and GS. The significances of the other three common algorithms (CCA-A, CCA-SNR, CCA-Band) and the GS are all greater than 0.05, indicating that the other three CCA related algorithms have no linear relationship with the GS. Further, the correlation coefficients between them are all less than 0.3, indicating that they are not correlated. It also further shows that USSR is more related to the GS than the other three common CCA algorithms in terms of fatigue difference.

**Table 2:** Pearson correlation test.

		USSR	CCA-A	CCA-SNR	CCA-Band
GS	Pearson correlation	0.724^**^	0.247	0.269	0.118
	Significance (bilateral)	0.002	0.374	0.333	0.676
	*N*	15	15	15	15

^**^p < 0.01.

Next, in order to further analyze the difference of the quantitative results of the five different algorithms in the two states of consciousness and fatigue, we dimensionless the fatigue increment of the five algorithms in the two states. The formula is as follows:


(6) $$FI=\frac{AQV\;\mathrm{at}\;F-AQV\;\mathrm{at}\;C}{AQV\;\mathrm{at}\;C}\times 100\%$$

Where FI denotes Fatigue increment, AQV represents algorithm quantification value, C means under consciousness state, F means under fatigue state. After obtaining the dimensionless fatigue increment of the five algorithms, the following analysis is performed.

Firstly, the Kolmogorov–Smirnov test is used to confirm the normality of the results of FI of the five visual fatigue algorithms. The results are as follows (Table 3).

**Table 3:** Kolmogorov–Smirnov test.

Algorithms	Significance	Results
GS	0.996	Normal distribution
USSR	0.272	Normal distribution
CCA-A	0.999	Normal distribution
CCA-SNR	0.269	Normal distribution
CCA-Band	0.374	Normal distribution

It can be seen that the five sets of data conform to the normal distribution, next, the homogeneity of variance is tested by the Levene test (Table 4).

**Table 4:** Homogeneity test of variances.

	Levene statistics	df1	df2	Significance
Based on mean	9.023	4	70	<0.001^***^
Based on median	8.069	4	70	<0.001^***^
Based on median and adjusted df	8.069	4	38.811	<0.001^***^
Based on trim mean	8.621	4	70	<0.001^***^

^***^p < 0.001.

The results show that the variance of the five groups of experimental data is uneven. Therefore, non-parametric methods are used for statistical testing. Since it is a comparison of multiple sets of data, Kruskal-Wallis H-test is used (Table 5).

**Table 5:** Kruskal-Wallis H-test.

Total *N*	75
Test statistic	46.794
Degree of freedom	4
Asymptotic significance (2-sided test)	<0.001^***^

^***^p < 0.001.

The above results indicate that the overall mean of each group is not equal, and pairwise comparison is required,

It can be seen from Table 6 that the three visual fatigue indicators related to CCA are significantly different from the results of the new algorithm (USSR) and the traditional subjective visual fatigue detection method (GS), and there is no significant difference between USSR and GS. This shows that the results of the three CCA-related fatigue algorithms are very different from the subjective GS results. However, from the perspective of objective quantification of EEG for visual fatigue, USSR can be well consistent with the subjective GS scores. This also proves that the objective quantification algorithm of visual fatigue proposed in this paper can well reflect the results consistent with the traditional subjective detection, and the accuracy of the data is better than the traditional CCA-based objective quantification algorithm of visual fatigue.

**Table 6:** Pairwise comparison of different algorithm results.

Algorithm 1–algorithm 2	Test statistic	Standard error	Standard test statistics	Significance	Adjust significance
A- SNR	−1.267	7.958	−0.159	0.874	1.000
A-Band	−4.533	7.958	−0.570	0.569	1.000
A-USSR	23.867	7.958	2.999	0.003^**^	0.027^*^
A-GS	44.667	7.958	5.613	<0.001^***^	<0.001^***^
SNR-Band	−3.267	7.958	−0.410	0.681	1.000
SNR-USSR	22.600	7.958	2.840	0.005^**^	0.045^*^
SNR-GS	43.400	7.958	5.454	<0.001^***^	<0.001^***^
Band-USSR	19.333	7.958	2.429	0.015^*^	0.151
Band-GS	40.133	7.958	5.043	<0.001^***^	<0.001^***^
USSR-GS	20.800	7.958	2.614	0.009^**^	0.090

^**^p < 0.01. ^***^p < 0.001. A denotes CCA-A, SNR denotes CCA-SNR, Band denotes CCA-Band.

## 4 Discussion

### 4.1 Advantages

It can be seen from the results that the visual fatigue assessment model induced by SSVEP paradigm based on the combination of SSVEP-based fixed-step energy parameter optimization and underdamped second-order stochastic resonance can improve the signal-to-noise ratio of EEG signals that objectively quantify visual fatigue, so as to more accurately reflect objective visual fatigue. There is a big difference between the quantitative degree of the traditional subjective method and the objective method. Therefore, compared with the traditional subjective method, the credibility of the current mainstream objective methods such as CCA is not strong. Compared with the traditional subjective and objective quantitative methods, the algorithm in this paper can better match the subjective fatigue degree of the subjects. There is no significant difference between the quantitative results and the visual fatigue degree of the subjective gold standard of the subjects, so it has stronger credibility. Through this quantitative model, we can express visual fatigue more intuitively and clearly.

At the same time, this model only needs single-channel EEG data. Compared with the multi-channel EEG data requirements of traditional quantitative models (Zhang et al., 2020; Gao et al., 2021; Huang et al., 2022), it can greatly reduce the acquisition cost and improve the user experience. The accurate and objective quantification of visual fatigue based on SSVEP paradigm can also be extended to the quantification of visual fatigue under the combination of different parameters of the display and different light environments, so as to provide more references for the optimization design of the relevant parameters of the display. In addition to visual fatigue, the use of EEG to detect traditional driving fatigue has also received more and more attention from the academic community (Min et al., 2021; Tuncer et al., 2021), and the corresponding objective quantification of brain fatigue can also be more accurately quantified using the method of this paper.

In order to demonstrate the universality of the model proposed in this manuscript, the following discussion is conducted. From the SSVEP data set named BETA in Tsinghua University (Liu et al., 2020), the data of the subjects with a stimulation frequency of 15.8 Hz is intercepted. Due to the individual differences in EEG data, some subjects with abnormal EEG data are excluded. On the basis of normal EEG data, the data of 11 Blocks of nine subjects is used, and compared the CCA amplitude (CCA-A) with the new algorithm (USSR) in this paper. It is found that even without changing the parameter selection range of the USSR, USSR can still improve the signal-to-noise ratio, so that visual fatigue could be quantified more obviously.

**Figure 7:**
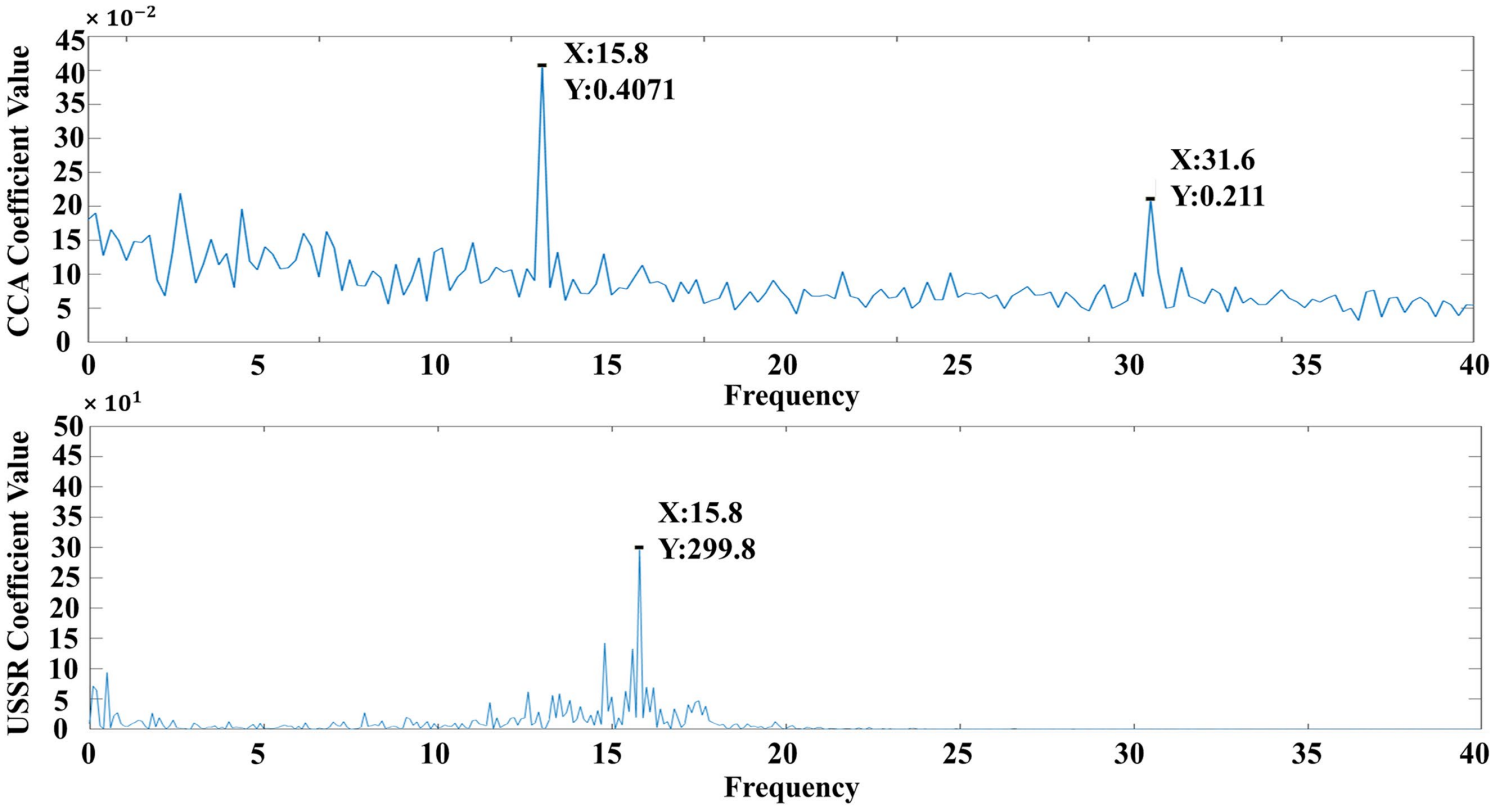
Difference between USSR and CCA in visual fatigue quantification based on EEG comparison.

**Figure 8:**
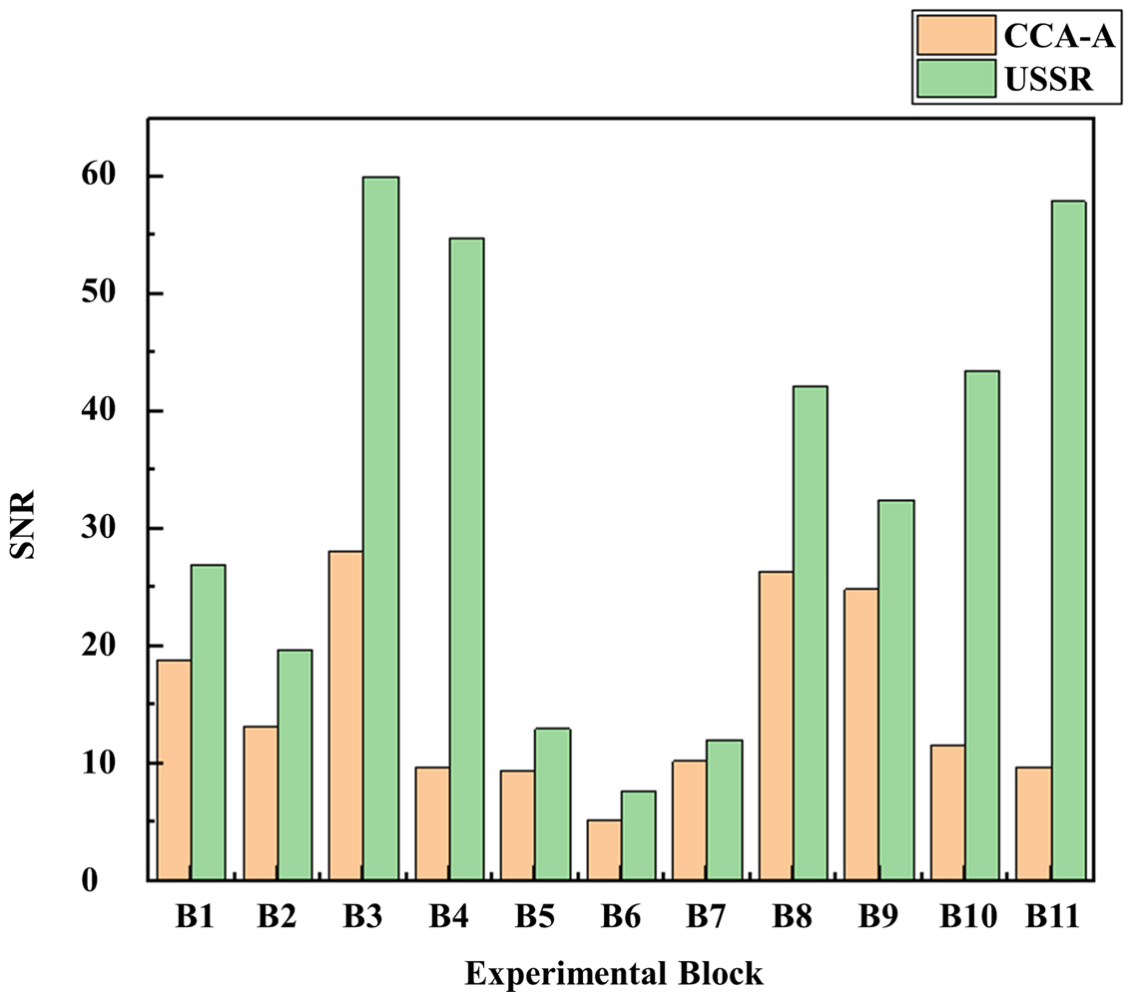
Difference between USSR and CCA-A in SNR based on EEG comparison.

It can be seen from Figures 7, 8 that the contrast effect of USSR and CCA at 15.8 Hz is similar to that of 7.5 Hz stimulation paradigm in the manuscript, which can improve the signal-to-noise ratio to a certain extent. It can be proved that compared with CCA-A (15.1689 ± 7.9674), USSR (33.6388 ± 19.2536) still has a good SNR improvement effect when the paradigm stimulation frequency is greater than 15 Hz. In summary, adding more stimuli frequencies from datasets above 15 Hz (higher frequencies have lower responses in SSVEP) so that the research would be validated for a wide range of visual fatigue analyzes.

### 4.2 Limitations

Although this model can better reflect the objective quantification of participants’ visual fatigue that induced by SSVEP paradigm, there is a limitation in this model, which is the parameter optimization method involved in the underdamped second-order stochastic resonance algorithm. The fixed step-energy parameter optimization model introduced in this paper can better solve this problem, but it takes a relatively long time, so only offline analysis can be carried out. Because of the high requirement for immediacy (Daly et al., 2015), the realization of online analysis needs more research. At present, there are two research directions. One is to find faster and more efficient parameter optimization algorithms, such as ant colony algorithm (Zheng et al., 2017; Wang Z. et al., 2022), genetic algorithm (Yom-Tov and Inbar, 2002; Garrett et al., 2003), etc., to replace the fixed range of parameter matrix, to achieve the purpose of finding the optimal parameter solution of the objective quantitative model proposed in this paper quickly and accurately; the other is to set more filter conditions in the data preprocessing so that the analysis process can meet the requirements of online analysis.

## 5 Conclusion

In modern society, electronic devices have entered thousands of households, and people‘s eye health is becoming more and more important. In this paper, a quantitative model for visual fatigue induced by SSVEP paradigm based on fixed-step energy parameter optimization and underdamped second-order stochastic resonance algorithm is proposed. Compared with the traditional related visual fatigue quantitative model, the subjective fatigue characteristics of the subjects can get a great degree of feedback. The model can be extended from the quantitative analysis of visual fatigue caused by SSVEP paradigm to the quantitative analysis of visual fatigue of electronic product users. Compared with traditional qualitative or quantitative analysis, the results obtained by this model are more in line with the real feedback of the experimenter. However, this model also has some shortcomings. On the one hand, the optimal selection of parameters takes a long time, especially since the analysis time caused by the different EEG data of the subjects is also different, so it is not suitable for online detection. On the other hand, after comparing the data of six channels, the model selects one channel as a sample for analysis, but we lack the data of other channels except these six channels. Therefore, the current channel selection may not be the best channel for objectively quantifying visual fatigue based on SSVEP. In summary, this model has greatly improved the objective quantification of visual fatigue compared with the existing methods, and the demand for the number of channels is low. It is an algorithm model that meets the needs of this direction.

## Data availability statement

The raw data supporting the conclusions of this article will be made available by the authors, without undue reservation.

## Ethics statement

All subjects were given informed consent according to the Helsinki Declaration and approved by the institutional review committee of Xi’an Jiaotong University. The studies were conducted in accordance with the local legislation and institutional requirements. The participants provided their written informed consent to participate in this study.

## Author contributions

PT: Data curation, Project administration, Resources, Software, Writing – original draft. GX: Conceptualization, Funding acquisition, Investigation, Methodology, Supervision, Writing – review & editing. CH: Formal analysis, Methodology, Validation, Visualization, Writing – review & editing. XuZ: Validation, Visualization, Writing – review & editing. XiZ: Conceptualization, Investigation, Methodology, Writing – review & editing. FW: Validation, Visualization, Writing – review & editing. SZ: Supervision, Validation, Visualization, Writing – review & editing. ZZ: Resources, Validation, Visualization, Writing – review & editing.
