# Supplementary material for: A quantization algorithm of visual fatigue based on underdamped second order stochastic resonance for steady state visual evoked potentials
Source: Front Neurosci. 2023 Nov 21;17:1278652. doi: 10.3389/fnins.2023.1278652 (PMC10702533; doi:10.3389/fnins.2023.1278652)
Supplement: Supplementary file 1 [file Table_1.DOCX]

Table 1

Twelve modes based on SSVEP paradigm

| Paradigm | Detailed Settings |
| --- | --- |
| 1 | 0% brightness – Green light flicker |
| 2 | 0% brightness – Blue light flicker |
| 3 | 0% brightness – Red light flicker |
| 4 | 0% brightness – Black light flicker |
| 5 | 50% brightness – Black light flicker |
| 6 | 50% brightness – Blue light flicker |
| 7 | 50% brightness – Green light flicker |
| 8 | 50% brightness – Red light flicker |
| 9 | 100% brightness – Blue light flicker |
| 10 | 100% brightness – Black light flicker |
| 11 | 100% brightness – Red light flicker |
| 12 | 100% brightness – Green light flicker |

Table 2

Pearson correlation test

|  |  | USSR | CCA-A | CCA-SNR | CCA-Band |
| --- | --- | --- | --- | --- | --- |
| GS | Pearson correlation | 0.724^**^ | 0.247 | 0.269 | 0.118 |
|  | Significance ( bilateral ) | 0.002 | 0.374 | 0.333 | 0.676 |
|  | N | 15 | 15 | 15 | 15 |

^**^P<0.01

Table 3

Kolmogorov-Smirnov test

| Algorithms | Significance | Results |
| --- | --- | --- |
| GS | 0.996 | normal distribution |
| USSR | 0.272 | normal distribution |
| CCA-A | 0.999 | normal distribution |
| CCA-SNR | 0.269 | normal distribution |
| CCA-Band | 0.374 | normal distribution |

Table 4

Homogeneity test of variances

|  | Levene statistics | df1 | df2 | Significance |
| --- | --- | --- | --- | --- |
| Based on mean | 9.023 | 4 | 70 | <0.001^***^ |
| Based on median | 8.069 | 4 | 70 | <0.001^***^ |
| Based on median and adjusted df | 8.069 | 4 | 38.811 | <0.001^***^ |
| Based on trim mean | 8.621 | 4 | 70 | <0.001^***^ |

^***^P<0.001

Table 5

Kruskal-Wallis H-test

| Total N | 75 |
| --- | --- |
| Test statistic | 46.794 |
| Degree of freedom | 4 |
| Asymptotic significance ( 2-sided test ) | <0.001^***^ |

^***^P<0.001

Table 6

Pairwise comparison of different algorithm results

| Algorithm 1 - algorithm 2 | Test statistic | standard error | Standard Test Statistics | Significance | Adjust significance |
| --- | --- | --- | --- | --- | --- |
| A- SNR | -1.267 | 7.958 | -0.159 | 0.874 | 1.000 |
| A-Band | -4.533 | 7.958 | -0.570 | 0.569 | 1.000 |
| A-USSR | 23.867 | 7.958 | 2.999 | 0.003^**^ | 0.027^*^ |
| A-GS | 44.667 | 7.958 | 5.613 | <0.001^***^ | <0.001^***^ |
| SNR-Band | -3.267 | 7.958 | -0.410 | 0.681 | 1.000 |
| SNR-USSR | 22.600 | 7.958 | 2.840 | 0.005^**^ | 0.045^*^ |
| SNR-GS | 43.400 | 7.958 | 5.454 | <0.001^***^ | <0.001^***^ |
| Band-USSR | 19.333 | 7.958 | 2.429 | 0.015^*^ | 0.151 |
| Band-GS | 40.133 | 7.958 | 5.043 | <0.001^***^ | <0.001^***^ |
| USSR-GS | 20.800 | 7.958 | 2.614 | 0.009^**^ | 0.090 |

^**^P<0.01, ^***^P<0.001, A denotes CCA-A, SNR denotes CCA-SNR, Band denotes CCA-Band
